# Correlation of the Plasma Concentration of Eltrombopag With Efficacy in the Treatment of Refractory Aplastic Anemia: A Single-Centre Study in China

**DOI:** 10.3389/fphar.2020.582625

**Published:** 2020-11-16

**Authors:** Wei Zuo, Bo Zhang, Jing Ruan, Miao Chen, Bing Han

**Affiliations:** ^1^ Department of Pharmacy, Peking Union Medical Colleague Hospital, Chinese Academy of Medical Science, Beijing, China; ^2^ Department of Hematology, Peking Union Medical Colleague Hospital, Chinese Academy of Medical Science, Beijing, China

**Keywords:** eltrombopag, non-severe aplastic anemia, refractory, plasma concentration, efficacy

## Abstract

**Background and purpose:** Eltrombopag (ELT) can be effective in the treatment of relapse/refractory aplastic anemia (AA) patients. Responses and adverse drug reactions (ADRs) differed greatly among individuals treated at the same dosage of ELT.

**Methods:** Patients diagnosed with nonsevere aplastic anemia (NSAA) between January 2018 and January 2019 in Peking Union Medical Colleague Hospital who were refractory to immunosuppressive therapy were treated with ELT and followed up for at least 6 months. Plasma concentrations of ELT were detected by high-performance liquid chromatography-mass spectrometry after at least two months of ELT treatment and treatment at the same dosage for at least 2 weeks. The dose-concentration, concentration-response and concentration-ADR relationships were evaluated.

**Results:** Among the 72 patients treated with ELT during the study period, 44 patients with complete data were enrolled. Six (13.6%) were males, and 38 were females (86.4%), with a median age of 54 years [interquartile range (IQR): 38.5–63]. At the time the ELT plasma concentration was detected, the median dosage of ELT was 75 (IQR 50–100) mg/d, the median time of total ELT exposure was 3 (IQR 2.0–6.0) months, and 37 (70.5%) patients had responded to ELT. The median concentration of ELT was 10.4 μg/ml (IQR 3.7–24.4 μg/ml). The concentration of ELT was positively correlated with the daily dose of ELT (*r* = 0.68, *p* < 0.001). Multivariate logistic regression analysis showed that the risk of inefficacy of ELT at a concentration between 11.2 and 15.2 μg/ml was 0.028-fold (95% CI: 0.001–0.864; *p* = 0.041) of that at a concentration between 3.2 and 7.2 μg/ml. The cutoff value for the concentration of ELT showing efficacy was 12.50 μg/ml according to the receiver operation characteristic curve. A higher risk of ADR was related to a longer total exposure to ELT (*p* = 0.012). Although the correlation was not significant, the odds ratio increased with the ELT concentration, suggesting that it was possible that an elevated risk of ADR was correlated with the ELT blood concentration.

**Conclusion:** ELT is effective for the treatment of NSAA and has acceptable side effects. The plasma concentration of ELT was correlated with the dose and the effects of ELT.

## Introduction

Few therapeutic options are available for patients with aplastic anemia (AA) who are ineligible for transplantation and refractory to immunosuppressive therapy. Eltrombopag (ELT), a small molecule thrombopoietin mimetic, has been proven to induce trilinear hematopoietic responses in relapsed/refractory AA patients (Desmond et al., 2014). The results of a phase II trial examining ELT monotherapy in refractory severe aplastic anemia patients showed a 40% response rate after 3–4 months of therapy (Olnes et al., 2012; Desmond et al., 2014), which led to the approval of ELT as a monotherapy for relapsed/refractory SAA in the United StatesA and Europe (Novartis, 2017; Novartis, 2018). However, the use of ELT is only approved for idiopathic thrombocytopenic purpura (ITP) in China by the National Medical Products Administration; for AA, the use of ELT is still “off-label” (Zhu et al., 2019). Therefore, it is not covered by national health insurance for the indication of AA. Currently, the use of ELT in Chinese patients with AA is still limited; moreover, the high price of ELT for long-term medication might further restrict its application.

Many studies have focused on the relationship between the dosage of ELT and its efficacy (Gibiansky et al., 2011; Matthys et al., 2011). However, the clinical responses and adverse drug reactions (ADRs) differed greatly among individuals, even those treated with the same dosage (Choy et al., 2016). To date, there have been few studies demonstrating the clinical significance of the blood concentration of ELT in patients with AA in daily clinical practice. Therapeutic drug monitoring is a branch of clinical pharmacology that specializes in the measurement of drug concentrations in blood, aiming at improving patient care by individually adjusting the dose of drugs (Oellerich et al., 2017). Early identification of populations for which a drug may be inappropriate might be helpful in adjusting the treatment plan and reducing the burden and expense. Given that the patients’ responses the to the drug differed dramatically, we would like to clarify whether plasma concentrations of ELT could be an indicator of its efficacy and/or adverse events.

## Materials and Methods

### Study Population

This is a prospective study. From January 2018 to January 2019, outpatients in Peking Union Medical Colleague Hospital (PUMCH, Beijing, China) diagnosed with nonsevere aplastic anemia (NSAA) and refractory to immunosuppressive therapy (cyclosporine A for at least 6 months) were treated with ELT. Cyclosporine A (CsA) had been stopped for at least 6 months before ELT treatment. No other drugs, such as tacrolimus, danazol, or stanozolol, were used during the experimental period. Blood samples from patients who had been treated with ELT for at least 2 months and had been treated at the same dosage for at least 2 weeks were included in the final analysis. The enrolled patients received at least four visits (once a month) to evaluate the efficacy and side effects. All participants and/or their legal guardians signed written informed consent forms before blood was drawn for tests. This study was approved by the Ethical Committee of PUMCH and was conducted in accordance with the Declaration of Helsinki.

### Clinical Data Collection

ELT was started at 25 mg/day for 2 weeks, and the dosage was increased every 2 weeks to a maximum of 100 mg/day and then tapered gradually (by 25 mg/day every 3 months) after the best response was achieved. Baseline information, including the clinical and laboratory data, was recorded. Symptoms (including side effects) and signs were recorded every month after treatment by one of the researchers, who was blinded to the outcome of the analysis. Laboratory assessments (complete blood count with differential and serum chemistry profiles, ECG or other tests if needed) were performed in our hospital and very occasionally in other local hospitals.

Patients were followed up for at least 6 months after ELT treatment, and the outcome was determined either from the medical documents or by telephone interviews of the patients or their relatives.

### Blood Sample Collection and Testing of the Plasma Eltrombopag Concentration

All patients enrolled had been exposed to ELT for at least 2 months and had been stable at the current dosage for 2 weeks to ensure a steady blood concentration. On the day of the blood draw, patients were asked to undergo ELT treatment under the supervision of the researchers to minimize the errors caused by time variation in the drawing of blood. Two hours later, blood samples were collected via the forearm vein into tubes containing K3-EDTA and were immediately placed in ice water. The plasma samples were separated and then stored at −80°C in polypropylene tubes until analysis. Plasma from healthy volunteers was obtained as a normal control.

The plasma concentrations of ELT were assayed by high-performance liquid chromatography-tandem mass spectrometry (Maddela et al., 2014). This assay can detect the entire range of ELT concentrations from 100 to 50,000 ng/ml. The imprecision and inaccuracy were less than 20 and 18%, respectively, whereas the intra-assay error was less than 10%. The ELT reference standard (99.69% pure) was obtained from MedChemExpress (United States, HY15306A). ELT 13C4 (99.66% pure) was employed as an internal standard and was obtained from TLC Pharmaceutical Standards Ltd.

### Criteria for Evaluation

Complete remission (CR) and partial remission (PR) were defined according to previous literature (Marsh et al., 2009). Briefly, 1) CR was defined as normal hemoglobin (HGB), neutrophils (ANC) >1.5 × 10^9^/L, and platelets (PLT) >100 × 10^9^/L; and 2) PR was defined as A) stoppage of blood transfusion (in patients who were previously blood transfusion-dependent); B) restoration of at least one lineage to normal or 2-fold increased compared with the baseline; and C) HGB >30 g/L (<60 g/L before treatment), ANC >0.5 × 10^9^/L (less than 0.5 × 10^9^/L before treatment), PLT >20 × 10^9^/L (less than 20 × 10^9^/L before treatment) compared with the baseline. No response (NR) was defined as not having any of the above responses.

The causality assessment of ADRs was performed with a standardized algorithm by two doctors based on the Common Terminology Criteria for Adverse Events Version 5.0 (U.S. Department of Health and Human Services, 2017).

### Statistical Analysis

Data are expressed as the median with the interquartile range (IQR) for continuous variables and as percentages/frequency (%) for categorical variables. Normally distributed data were compared by Student's t-test, while non-normally distributed data were compared by the Mann–Whitney U test. Categorical data were analyzed by the chi-square test. Logistic regression analysis was conducted to determine the relationship between ELT efficacy or adverse drug reactions (ADRs) and ELT concentrations by adjusting for potential confounding factors. *p*-values and 95% CIs were two-sided. *p* < 0.05 was considered statistically significant. Data were analyzed using SPSS version 17.0.

## Results

### Patient Demographics

Very few patients with SAA were treated with ELT. To reduce the heterogeneity of the patient population, we focused only on patients with NSAA. From January 2018 to January 2019, 72 patients with NSAA who were refractory to CsA received ELT treatment. Among them, 10 patients were exposed to ELT for less than 2 months and were switched to other medications for various reasons. Eight patients refused to give blood for ELT concentration determination, and 10 patients were lost to follow-up within 6 months of ELT treatment. Overall, 44 patients were included in the final analysis, with a median age of 54 years (IQR: 38.8–63.0). Of the 44 patients, 6 (13.6%) were males, while 38 were females (86.4%). To avoid selection bias, we double-checked the data of the included and excluded patients. There were no significant clinical, hematological or treatment differences between the screened and included patients. Before the initiation of the ELT regimen, patients had been treated with CsA for a median of 12 months (6–36), and the rate of transfusion was 2 (0–6) and 2 (0–7) units/month for red blood cells and 2 (0–4) and 2 (0–4) for platelets at the time of diagnosis and ELT treatment initiation, respectively. The median hemoglobin count was 69.0 g/L (IQR 56.3–91.0 g/L), with a median platelet count of 9.5 × 10^9^/L (IQR 6.8–14.0 × 10^9^/L), a median absolute neutrophil count of 1.16 × 10^9^/L (IQR 0.8–1.6), and a median reticulocyte count of 51.5 × 10^9^/L (IQR 47.1–71.6) at the time of ELT treatment initiation. At the time blood was obtained for concentration determination, the median dosage of ELT was 75 (IQR 50.0–100.0) mg/d, and the median time of total ELT exposure was 3 (IQR 2.0–4.0) months. Accordingly, the median hemoglobin count at the same time was 80.5 (IQR 65.0–105.0) g/L, with a median platelet count of 26.0 (IQR 14.0–19.0) × 10^9^/L and a median absolute neutrophil count of 1.6 (IQR 1.6–1.7) × 10^9^/L. All the baseline clinical characteristics are shown in Table 1.

**TABLE 1:** Clinical characteristics of the patients enrolled.

	Total	Response group	Non-response group	*p*
Number of patients (n, %)	44 (100.0)	31 (70.5)	13 (29.5)	—
Demographic characteristics				
Gender, n. (%)^a^				0.83
Male	6.0 (13.6)	4.0 (12.9)	2.0 (15.4)	—
Female	38 0.0 (86.4)	27.0 (87.1)	11.0 (84.6)	—
Age, years, [IQR]^b^	54.0 [38.5, 63.0]	54.0 [38.5, 62.0]	53.0 [39, 64.0]	0.680
Weigh, kg [IQR]^b^	63.8 [56.5, 67.0]	65.0 [57.5, 68.5]	60.0 [55.3, 65.5]	0.25
Height, cm [IQR]^b^	163.0 [160.0, 165.0]	162.5 [160.0, 165.0]	163.0 [160.0, 165.0]	0.63
Previous CsA median months (range)	12 [6, 36]	11 [6, 32]	14 [7, 36]	0.73
Red blood cells (U/month)				
Time on diagnosis	2 [0–6]	2 [0–4]	2 [0–6]	0.91
Time on ELT onset	2 [0–7]	2 [0–6]	2 [0–7]	0.81
Platelet (U/month)				
Time on diagnosis	2 (0–4)	2 [0–3]	2 [0–4]	0.83
Time on ELT onset	2 (0–4)	2 [0–4]	2 [0–4]	0.96
Biological characteristics				
Before ELT				
Hemoglobin, g/L [IQR]^c^	69.0 [56.3, 91.0]	70.0 [50.5, 89.0]	65.0 [57.0, 91.0]	0.57
Neutrophils, absolute count, ×10^9^/L [IQR]^c^	1.2 [0.8, 1.6]	1.2 [0.8, 1.7 ]	1.1 [1.0, 1.4]	0.69
Platelet count, ×10^9^/L [IQR]^c^	9.5 [6.8, 14.0]	9.0 [7.0, 13.0]	10.0 [6.0, 19.0]	0.78
Reticulocyte counts, ×10^9^/L [IQR]^c^	51.5 [47.1.71.6]	52.0 [46.5,70.5]	51.5 [50.2, 71.6]	0.88
PNH Clone (n, %)^a^				0.95
No PNH clone	41.0 (93.2)	29.0 (93.5)	12.0 (92.3)	—
With small PNH clone	3.0 (6.8)	2.0 (6.5)	1.0 (7.7)	—
SGPT, U/L, [IQR]^c^	47.0 [22.0.92.0]	39.0 [16.5.88.5]	52.0 [32.0,110.0]	0.45
TBIL, μmol/L, [IQR]^c^	7.2 [3.9, 11.1]	5.9 [2.8.7.8]	7.1 [7.2.10.8]	0.39
Scr, μmol/L, [IQR]^c^	59.5 [26.0–152.0]	45.0 [26.0.97.0]	69.0 [34.0,105.0]	0.31
LDH, U/L, [IQR]^c^	262.0 [209.0,328.0]	304.0 [271.5,354.0]	257.0 [207.5,262.0]	0.59
SF, ng/ml, [IQR]^c^	573.0 [53.8,1423.5]	637.0 [35.0,3783.0]	502.0 [65.0,2782.0]	0.41
At the time of blood taken for concentration				
Hemoglobin, g/L [IQR]^c^	80.5 [65.0,105.0]	83.0 [70.0,107.5]	79.0 [61.0,101.0]	0.32
Neutrophils, absolute count, ×109/L [IQR]^c^	1.6 [1.6.1.7]	1.8 [1.3.2.2]	1.1 [1.1.1.7]	0.04
Platelet count, ×109/L [IQR]^c^	26.0 [14.0.49.0]	40.5 [17.0.84.5]	14.0 [9.0.26.0]	0.00
Reticulocyte counts, ×10^9^/L [IQR]^c^	51.5 [50.1.70.3]	51.0 [49.5,71.5]	51.5 [50.0, 70.1]	0.86
White blood cell, absolute count, ×109/L [IQR]^c^	3.4 [2.9–4.2]	3.4 [2.9.3.9]	3.6 [3.0.4.3]	0.71
Mean corpuscular volume, fL [IQR]^c^	105.2 [98.4,111.1]	105.9 [97.9,112.3]	102.0 [100.0,107.3]	0.24
Lymphocytes, absolute count, ×109/L [IQR]^c^	1.4 [2.0.1.0]	1.2 [1.1.1.8]	2.1 [0.9.2.8]	0.15
Transaminases, n (%)^a^				
Reduced	3.0 (6.8)	3.0 (9.7)	0 (0)	0.68
Normal	24.0 (54.5)	16.0 (51.6)	8.0 (61.5)	—
Elevated	17.0 (38.6)	12.0 (38.7)	5.0 (38.5)	—
Serum creatinine, n. (%)a				
Normal	34.0 (77.3)	23.0 (74.2)	11.0 (84.6)	0.37
Elevated	10.0 (22.7)	8.0 (25.8)	2.0 (15.4)	—
ELT at the time of blood concentration				
Months from ELT start [IQR]^c^	3.0 [2.0.4.0]	3.0 [2.0.4.0]	2.3 [2.0.4.0]	0.97
Weeks with the current dose [IQR]^c^	3.0 [2.0.4.0]	3 [2.0.4.0]	3 [2.0.4.0]	1.00
Patients with different dose, n (%)^a^	75.0 [50.0.75.0]	75.0 [50.0.75.0]	75.0 [50.0.75.0]	1.00
25 mg/d	3.0 (6.8)	2.0 (6.4)	1.0 (7.7)	—
50 mg/d	14.0 (31.8)	10.0 (32.3)	4.0 (30.8)	—
75 mg/d	17.0 (38.6)	12.0 (38.7)	5.0 (38.5)	—
100 mg/d	10.0 (22.7)	7.0 (22.6)	3.0 (23.1)	—
Plasma concentration, μg/ml				
Min, Max	3.7, 24.4	3.7, 24.4	5.2, 20.5	—
Median [IQR]^c^	10.4 [8.2.13.8]	12.0 [8.8.14.6]	9.5 [8.1–10.4]	0.13
Graded concentrations, n (%)^a^				0.08
[3.2–7.2] μg/ml	8.0 (18.2)	5.0 (16.1)	3.0 (23.1)	—
[7.2–11.2] μg/ml	15.0 (34.1)	8.0 (25.8)	7.0 (53.8)	—
[11.2–15.2] μg/ml	13.0 (29.5)	11.0 (35.5)	2.0 (15.4)	—
[>15.2] μg/ml	8.0 (18.2)	7.0 (22.6)	1.0 (7.7)	—
ELT follow-up time, month, [IQR]^c^	8.0 [7.0.10.0]	9.0 [7.0.10.0]	8.0 [6.0, 11.5]	0.80

IQR, interquartile range; AA, aplastic anemia; CsA, cyclosporine; CR, Complete remission; PR, partially remission; OR, overall remission; SGPT, serum glutamic-pyruvic transaminase; TBIL, total bilirubin; Scr, serum creatinine; LDH, lactate dehydrogenase; SF, serum ferritin. Comparisons of variable between the different concentration groups.

a Pearson’s chi-square test.

b Student’s t-test.

c Nonparametric Mann–Whitney test.

### Eltrombopag Treatment and Response

Patients were followed up for a median of 8 (IQR: 7.0–10.0) months, and the longest follow-up period was 12 months after ELT treatment. According to their response at the time of blood concentration determination, patients were divided into the response group (those who had achieved at least PR) and the nonresponse group (patients with NR). There was no difference in the baseline characteristics in the groups with or without response (Table 1). Moreover, the total time of exposure to ELT and the time at the current dosage were the same between the two groups (*p* = 0.37 and *p* = 0.54, respectively).

**FIGURE 1:**
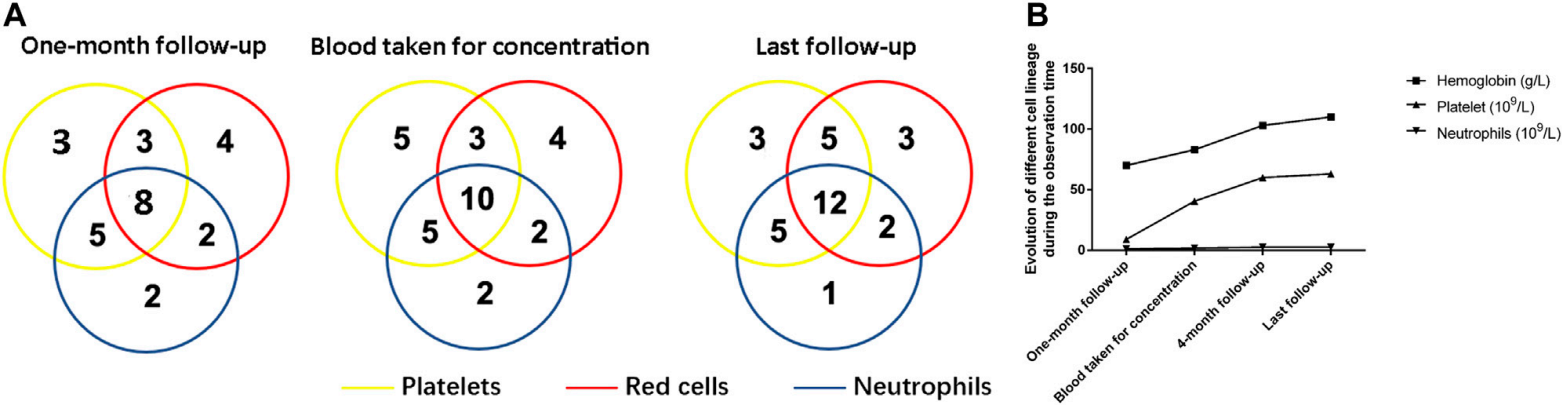
Hematologic improvements caused by Eltrombopag at different time points. **(A)** The Venn diagrams show the number of patients with unilineage, bilineage, and trilineage hematologic responses at the time when the blood concentration was determined and at the time of last follow-up. **(B)** A cumulative line chart showing hemoglobin/platelet/neutrophil evolution during the observation time.

One month after ELT, an overall response was observed in 61.4% (27/44) of patients. The response rate increased over time. By the end of the follow-up period, one patient with NR had achieved PR, and two patients with PR had achieved CR, whereas one patient with PR relapsed when ELT was still being used, resulting in final odds ratio (OR), CR and PR rates of 70.5% (31/44), 27.3% (12/44), and 43.2% (19/44), respectively. The hematologic cell lineage response at different time points is shown in Figure 1. Three patients stopped ELT treatment due to a lack of effectiveness or relapse at 8, 12, and 12 months. No death or clone evolution was noticed during the follow-up period.

### Clinical Significance of the Plasma Concentration of Eltrombopag

**FIGURE 2:**
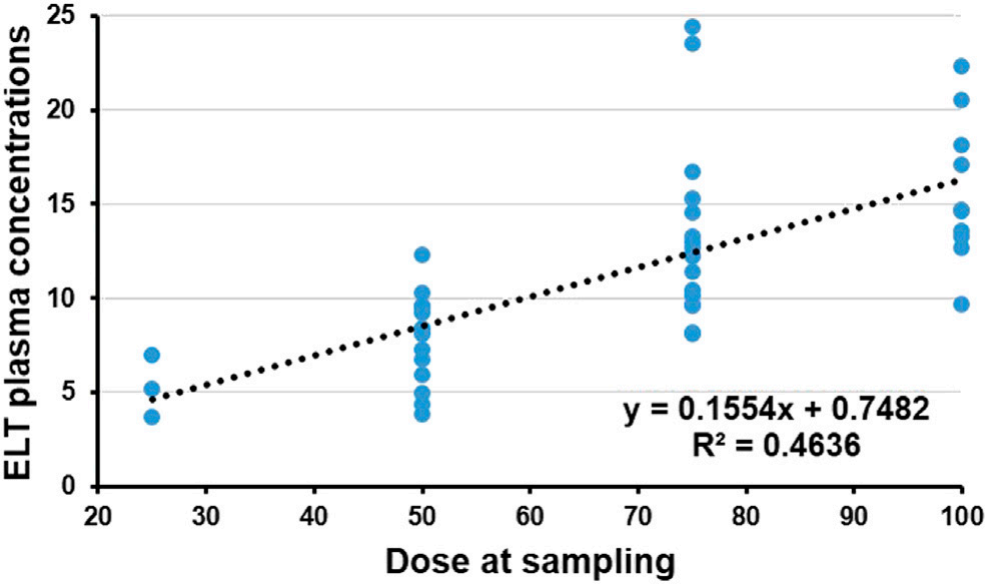
Plasma concentration and dose of Eltrombopag (ELT). The regression equation of the relationship between the dose of ELT and concentration was successfully determined (*Y* = 0.7482 + 0.1554*X*, *p* < 0.001). The plasma concentration of ELT was significantly positively correlated with the daily dose of ELT (*r* ^2^ = 0.4636, *p* < 0.001).

All enrolled patients were tested to determine the plasma concentration 2 h after receiving ELT (peak concentration). The median concentration of ELT was 10.4 μg/ml (IQR 3.7–24.4 μg/ml). Potential clinical characteristics that may have influenced the concentration of ELT were summarized and calculated in the multivariate regression analysis (Table 2). The regression equation showing the relationship between the ELT dose and concentration was successfully determined (*Y* = 0.7482 + 0.1554*X*, *p* < 0.00), and the blood concentration of ELT was positively correlated with the daily dose of ELT (*r* = 0.68, *p* < 0.001, Figure 2). We also found a positive relationship between the ELT plasma concentration and the magnitude of platelet improvement (*p* < 0.05) and lymphocyte improvement (*p* < 0.05), as shown in Figure 2. Since no patients had severe impairment of kidney or liver function after ELT, no relevant significant change in the plasma concentration was noticed in our study, as indicated in Table 1. No correlation between the plasma concentration of ELT and other clinical characteristics was found (Table 2).

**TABLE 2:** Correlations of clinical characteristics and plasma concentrations of ELT.

Number	Pearson’s *r*	*p*
Demographic characteristics		
Age, years	0.08	0.23
Weigh, kg	0.11	0.67
Height, cm	0.05	0.38
Gender	−0.11	0.24
Biological characteristics		
Before ELT		
Hemoglobin, g/L	−0.42	0.67
Neutrophils, absolute count, x10^9^/L	−0.10	0.68
Platelet count, x10^9^/L	−0.24	0.20
Transaminases	−0.00	0.88
Serum creatinine	0.25	0.86
Magnitude of hematological improvement (at the time of TDM)		
Neutrophils, absolute count, ×10^9^/L	0.11	0.41
Platelet count, ×10^9^/L	0.58	0.04
Lymphocytes, absolute count, ×10^9^	0.51	0.05
ELT treatment		
Months from ELT started	0.16	0.36
Months with the current dose	0.27	0.08
Dosage, mg/day	0.68	0.00

### The Correlation Between the Plasma Concentration and the Efficacy of Eltrombopag

**FIGURE 3:**
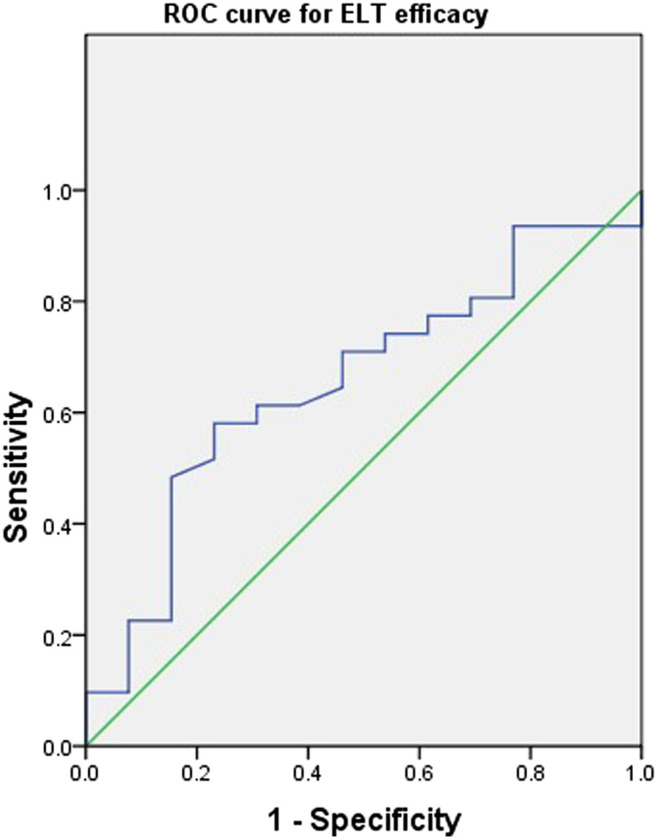
Receiver operating characteristic curves of the Eltrombopag concentration and efficacy for 44 patients with refractory nonsevere aplastic anemia. A plot of test sensitivity (*y* coordinate) vs. the false positive rate (*x* coordinate) was obtained at each cutoff level. The area under the smooth ROC curve and its 95% CI were 0.65 and 0.48–0.83, respectively.

Due to the small number of patients, individual variables with a *p*-value less than 0.20 in Table 1 were included in the following multivariate regression analysis. After identifying other influencing factors with a multivariate logistic regression analysis, a significant correlation between ELT efficacy and concentration (Table 3) was found. The OR decreased as the concentration of ELT increased, suggesting an elevated response rate at a higher ELT plasma concentration. A concentration between 11.2～15.2 μg/ml was associated with a 0.03-fold (95% CI, 0.00–0.86; *p* = 0.04) lower risk of ineffectiveness compared with the reference concentration. However, when the concentration of ELT was above 15.2 μg/ml, the response rate was slightly decreased compared to that associated with concentrations between 11.2 and 15.2 μg/ml. To determine the cut-off values of the relationship of the ELT concentration to efficacy, ROC curves were generated with AUCs of 0.65 (95% CI, 0.48–0.83) (Figure 3). The diagnostic specificity of ELT inefficacy increased, and the sensitivity decreased with increasing ELT concentration. Youden's index identified the optimal cut-off point as 12.5 μg/ml. At this threshold, the sensitivity was 51.6%, and the specificity was 84.6%.

**TABLE 3:** Multivariate logistic regression analysis of variables associated with inefficacy.

Covariate	Ineffective	
	OR (CI)	*p*-value^a^
Graded concentration, μg/ml		
[3.2–7.2]	Ref	—
[7.2–11.2]	0.33 [0.02, 7.10]	0.48
[11.2–15.2]	0.03 [0.00, 0.86]	0.04*
[>15.2]	0.09 [0.00, 3.18]	0.19
Biological characteristics (at the time of TDM)		
Neutrophils, absolute count, ×10^9^/L	1.15 [0.91, 1.44]	0.24
Platelet count, ×10^9^/L	0.87 [0.76, 0.99]	0.04*
Lymphocytes, absolute count, ×10^9^	7.85 [0.93, 66.49]	0.06

OR: odd ratio; Ref, reference category for the odds ratio.

a *p*-value calculated using multivariate regression analysis.

*p < *0.05*.

### Correlation Between the Plasma Concentration of Eltrombopag and Adverse Drug Reactions

Adverse effects were reported in 17/44 (38.6%) patients. The two most common ADRs reported were lower limb oedema (11.4%, 5/44) and the elevation of transaminase (6.8%, 3/44). Other reported ADRs included skin rash (4.5%, 2/44), creatine elevation (4.5%, 2/44), nausea/vomiting (2.3%, 1/44), skin segmentation (2.3%, 1/44), tachycardia (2.3%, 1/44), oropharyngeal blister (2.3%, 1/44) and musculoskeletal pain (2.3%, 1/44). All ADRs were grade one. Multivariate logistic regression analysis was performed to determine the possible risk factors for ADRs, including gender, age, ELT concentration, renal/liver function and other biological characteristics. From the results, we can see that a higher risk of ADRs was related to a longer total exposure time to ELT (*p* = 0.01). Although no significant relationship was noticed between ADRs and the ELT concentration (*p* > 0.05), the OR increased with the ELT concentration, suggesting a possible elevation of the risk of ADRs with the blood concentration (Table 4). No significant relationships were noticed for other risk factors.

**TABLE 4:** Multivariate logistic regression analysis of variables associated with adverse drug reaction.

Covariate	Adverse effect	
	OR (CL)	*p*-value^a^
Gender	1.861 [0.155, 22.344]	0.24
Age	1.023 [0.696, 1.080]	0.41
Months from ELT started	1.307 [1.060, 1.611]	0.01*
Transaminases	0.167 [0.102, 98.362]	0.79
Serum creatinine	1.192 [0.162, 7.725]	0.86
Graded concentration, μg/ml		
[3.2–7.2]	1	0.64
[7.2–11.2]	7.07 [0.22, 226.13 ]	0.27
[11.2–15.2]	23.77 [0.58, 971.45]	0.09
[>15.2]	5.37 [0.13, 218.35]	0.37

OR, odd ratio; Ref, reference category for the odds ratio.

a *p*-value calculated using multivariate regression analysis.

* *p* < *0.05*.

## Discussion

In this study, we selected patients with refractory NSAA who had been treated with ELT for at least 2 months to evaluate the correlation between the plasma concentration and the efficacy of ELT. Those who received ELT for less than 2 months or who withdrew within 2 months of ELT treatment initiation for different reasons (economic insufficiency, NR, side effects, lost follow-up, etc.) were excluded. Some patients refused to sign the consent form for blood testing, especially those with a poor response, which may partly explain the relatively high response rate (70.5%) in our cohort compared to that in the literature (Olnes et al., 2012; Desmond et al., 2014; Lengline et al., 2018; Ecsedi et al., 2019). Although the response rate was calculated at the time when blood was obtained to determine the ELT concentration, which may have been too short for an efficacy evaluation, most of the patients had already been exposed to ELT for more than 3 months in our cohort. It has been shown in the literature that most refractory SAA patients responded to ELT within 3 months of treatment initiation (Olnes et al., 2012). Moreover, the follow-up data of such patients showed that only a few showed an increase or decrease in efficacy subsequently, making the time of the blood concentration test a good time for determining the efficacy of ELT. Similar to reports from other investigators, nearly half of the patients had a trilineage response rather than only a platelet response (Desmond et al., 2014).

Although other studies have verified the effectiveness of ELT for refractory AA (Lengline et al., 2018), the optimal dosage and treatment duration have not been well defined so far. To the best of our knowledge, no data are available for determining the concentration–effect relationship of ELT. In most cases, increasing the dosage is an easy solution for patients with a poor response, but this may lead to a high economic burden or additional side effects. All enrolled patients had been exposed to ELT for at least 2 months and were stable at the current dosage for 2 weeks to ensure a steady blood concentration; furthermore, patients were tested to determine the peak concentration (2 h after ELT) to minimize the errors caused by variations in the time of the blood draw. Our results showed that the blood concentration of ELT was positively correlated with the daily dose of ELT within a certain range of concentrations. Under these circumstances, increasing the dose can increase the concentration of ELT and thus improve the efficacy. However, it seemed that the response rate was slightly decreased in patients with ELT concentrations above 15.2 μg/ml compared with that in patients with concentrations between 11.2 and 15.2 μg/ml, illustrating the minimal benefit of substantially increasing the dosage. In addition, we calculated a cut-off point of 12.50 μg/ml for optimal sensitivity and specificity. Although they need to be verified in further studies, these results implied that there is a dose-effect correlation for ELT in the treatment of refractory NSAA.

On the other hand, it has been reported previously that Asians may show slower metabolism of ELT compared with other ethnic groups (Wu et al., 2015), which may suggest that a lower dosage of ELT be used in Asian patients. However, a recent report from Hong Kong showed that higher doses (up to 300 mg) were tolerable and effective for Chinese patients, although some significant skin pigmentation was observed in patients treated with high doses (Gill et al., 2017; Hwang et al., 2018). However, a few other reports from Japan showed that a median dose of 84 mg (25–100 mg) can achieve an efficacy of 48%–55% (Konishi et al., 2019). In our study, a response could be observed at relatively low doses of 50–100 mg/day and at a median of 3 months. None of the patients received a dosage greater than 100 mg/day. However, the OR rate was 70.5%, and most of the patients reached the peak concentration that may indicate a favourable response, as shown in our study. We did not monitor the ELT concentration dynamically because the patients were unwilling to undergo frequent blood draws. Only four patients had their ELT concentration rechecked three times during their follow-up period. Three of them had low concentrations the first time blood was obtained and had a poor response at the same time. Neither an increase in their ELT concentration nor an improvement in their response was noticed after a longer period of ELT exposure (checked at 3, 6, and 8 months, respectively) at the same dosage. However, one patient had a high concentration (75 mg/d, 14.2 μg/ml) the first time blood was obtained and had a good response to ELT. He maintained a good response after dosage reduction to a final dosage of 50 mg/d, and the concentrations at 6 and 8 months were 11.2 μg/ml and 11.8 μg/ml, respectively. Those patients with a lower concentration and a poor response the first time blood was obtained for concentration determination did not show an increase in their ELT concentration or an improvement in their response after exposure to ELT for a longer time when the dose of ELT remained the same, suggesting that the 2 months duration of our experiment may be long enough to predict the effectiveness of ELT at that dosage. Collectively, our data indicated that, similar to CsA concentration monitoring in the treatment of AA (Liang et al., 2011; Philippe et al., 2015), monitoring of the concentration of ELT at certain points might also be helpful for the prediction of effectiveness.

Some factors, such as residual hematopoiesis, telomere length, age, degree of bone marrow hypocellularity, megakaryocytic morphology (absent megakaryocytes vs. reduced megakaryocytes) and even the polyclonal lymphoid marrow percentage, have been demonstrated as predictors of the response to ELT (Desmond et al., 2014; Hwang et al., 2018; Fattizzo et al., 2019). The baseline characteristics were compatible for patients with or without a response in our cohort, which was probably due to the small number of patients and the high lost to follow-up rate, which made our patient population highly selected. Nevertheless, the plasma concentration of ELT might be a potential reference for determining the response as well, as shown in our results.

Elevated transaminase is the most common dose-dependent toxic effect of ELT (Ecsedi et al., 2019), according to published data. Other side effects mentioned in the literature include skin rash, elevated bilirubin, skin pigmentation, nausea, fatigue, cough, diarrhea, headache, and possible clone evolution (Lum and Grainger, 2016; Scheinberg, 2019). In a recent study in Europe, ADRs were reported in 28% (51/180) of patients (Ecsedi et al., 2019). It has been shown that high-dose or long-term use of ELT may cause a higher rate of ADRs (Yang et al., 2017). In our study, approximately 38.6% of patients (17 in 44) reported ADRs. In accordance with the literature, a higher risk of ADRs was related to a longer total exposure time to ELT. Although the OR seemed to increase with the ELT concentration, suggesting a possible elevated risk of ADR, most of the ADRs were mild and easily controlled without drug suspension. This was probably because all the patients in our study were treated with a relatively low dose of ELT compared with that used in other reports, suggesting that a low dose range may be safer and more effective for Chinese patients.

Our study has some limitations. This was a single-centre study with a relatively small sample size, which might contribute to the uneven distribution of the male/female ratio in our cohort. The patients were not successive, and there might have been selection bias. The loss to follow-up rate was high, which might cause a higher OR rate since patients with poor responses or severe side effects may have been lost. The lack of dynamic monitoring of the concentration and the response rate might limit the evaluation of long-term efficacy and ADRs. However, our study demonstrated the efficacy of ELT in patients with refractory NSAA with acceptable toxicity.

Our study showed that the blood concentration of ELT within a certain range was correlated with the dose of ELT and probably indicated the OR rate. However, a high drug concentration exceeding a certain value might not further improve the efficacy. Long-term exposure to ELT may lead to a higher risk of ADR. The blood concentration of ELT might be associated with ADRs, but the lower and upper limits of the dose of ELT applied in our study might have protected against ADRs. Further studies with a larger number of patients and longer follow-up times with dynamic monitoring are needed.

## Data Availability Statement

The raw data supporting the conclusions of this article will be made available by the authors, without undue reservation.

## Ethics Statement

The studies involving human participants were reviewed and approved by Ethical Committee of the Peking Union Medical College and Chinese Academy of Medical Sciences. The patients/participants provided their written informed consent to participate in this study.

## Author Contributions

Drug concentration test: WZ and BZ; Clinical data collection: WZ, JR, and MC; Project administration, BZ; Supervision, BH; Writing–original draft: WZ; Writing–review and editing: BH.

## Funding

This study was supported by grants from Beijing Natural Science Foundation (7192168)，the Chinese Academy of Medical Sciences (CAMS) innovation fund for medical sciences (2016-I2M-3-004), the Nonprofit Central Research Institute Fund of Chinese Academy of Medical Sciences (2019XK 320047), and the National Natural Science Foundation of China Grants (81974183).

## Conflict of Interest

The authors declare that the research was conducted in the absence of any commercial or financial relationships that could be construed as a potential conflict of interest.
